# Prediction Model for Hemorrhagic Complications after Laparoscopic Sleeve Gastrectomy: Development of SLEEVE BLEED Calculator

**DOI:** 10.1007/s11695-016-2417-4

**Published:** 2016-10-11

**Authors:** Michal R. Janik, Maciej Walędziak, Jakub Brągoszewski, Andrzej Kwiatkowski, Krzysztof Paśnik

**Affiliations:** Department of General, Oncologic, Metabolic, and Thoracic Surgery, Military Institute of Medicine, Szaserów 128 Street, 04-141 Warsaw, Poland

**Keywords:** Sleeve gastrectomy, Hemorrhagic complications, Bleeding, Risk model, Risk calculator, Sleeve bleed, Complications

## Abstract

**Introduction:**

Laparoscopic sleeve gastrectomy (LSG) is one of the most frequently performed bariatric procedures. Hemorrhagic complications (HC) after surgery are common and require surgical revision. Accurate estimation of the risk of postoperative HC can improve surgical decision-making process and minimize the risk of reoperation. The aim of the present study was to develop a predictive model for HC after LSG.

**Material and Methods:**

The retrospective analysis of 522 patients after primary LSG was performed. Patients underwent surgery from January 2013 to February 2015. The primary outcome was defined as a surgical revision due to hemorrhagic complications. Multiple regression analysis was performed.

**Results:**

The rate of hemorrhagic complications was 4 %. The mean age of patients was 41.0 (±11.6) years and mean BMI was 47.3 (±7.3) kg/m^2^. Of the 12 examined variables, four were associated with risk of HC. Protective factors for HC were no history of obstructive sleep apnea (odds ratio [OR] 0.22; 95 % CI 0.05–0.94) and no history of hypertension (OR 0.38; 95 % CI 0.14–1.05). The low level of expertise in bariatric surgery (OR 2.85; 95 % CI 1.08–7.53) and no staple line reinforcement (OR 3.34; 95 % CI 1.21–9.21) were associated with higher risk of HC.

**Conclusions:**

The result revealed the association between hemorrhagic complications and the following factors: obstructive sleep apnea, hypertension, level of expertise in bariatric surgery, and reinforcement of the staple line. The risk assessment model for hemorrhagic complications after LSG can contribute to surgical decision-making process.

## Introduction

Bariatric surgery is very popular worldwide. Laparoscopic sleeve gastrectomy (LSG) is one of the most frequently performed bariatric procedures [1]. LSG constitutes of 60 % of all bariatric procedures in Poland [2]. This procedure is effective and relatively simple [3–5]. However, it is associated with a risk of serious surgical complications including gastric fistulas and hemorrhagic complications (HC). The incidence of HC after LSG is up to 4.94 % [6]. More than 3 % of patients require reoperation after LSG [7]. Most researchers have focused on gastric leakage and have neglected the issue of HC. Accurate estimation of the risk of postoperative HC can improve surgical decision-making process and minimize the risk of reoperation. Surgeons and patients would also benefit from the identification of factors that are associated with an increased risk of postsurgical HC. The previously published predictive mortality risk models in bariatric surgery were limited by the wide spectrum of outcomes and included different types of procedures [8–10]. In only one case was the risk model dedicated to the LSG procedure [11]. Therefore, the aim of the present study was to develop a predictive model for HC after LSG.

## Methods

The data of 552 patients were retrospectively collected from medical records. Obese patients undergoing LSG as a primary bariatric procedure from January 2013 to February 2015 were included. Patients undergoing revisional bariatric procedures were excluded. The primary outcome was surgical revision due to HC, which included bleeding and the presence of large hematomas on ultrasound examination. Bleeding was diagnosed on the basis of vital signs, including tachycardia (>120 beats per minute), hypotension (<90/60 mmHg), and clinical exam, including abdominal pain and drainage type. In those cases, we initiated fluid resuscitation and performed laboratory tests, as well as ultrasound examination. Patients with a poor response to fluid resuscitation, or the presence of free fluid, or hematoma in the peritoneal cavity on ultrasound examination were returned to the operating room for diagnostic laparoscopy.

The independent demographic variables were sex, age, and body mass index. Examined comorbidities included diabetes, hypertension, obstructive sleep apnea, dyslipidemia, hypothyroidism, and hyperthyroidism. Several surgical factors were also considered as follows: the surgeon’s level of expertise (>50 laparoscopic bariatric surgeries per year), the surgeon’s qualifications (senior vs. resident), and staple line reinforcement (running suture vs. none).

### Description of Procedure and Postoperative Management

The five-trocar technique was used. The gastrocolic ligament was cut to the angle of His with an energy device, and the diaphragmatic crura were exposed. A 36-French probe was used to calibrate the sleeve. A laparoscopic stapler was introduced and fired consecutively along the length of the bougie. We used two different staplers: (1) the Echelon Flex™ Endopath® stapler (Ethicon Endosurgery, Inc., Somerville, NJ, USA) with gold cartridges and (2) the Endo GIA™ (Covidien/Medtronic, Inc., Mansfield, MA, USA) with purple cartridges for the first two firings and blue cartridges for the remainder. Oversewing of gastrointestinal staple lines was used as a method for reinforcement. The decision of reinforcement was dependent upon the surgeon and was mainly influenced by intraoperative blood ooze.

In the oversewing group, the entire staple line was reinforced with a continuous extraserosal invaginating suture using 3–0 Biosyn™ Monofilament Absorbable Suture (Medtronic Inc., Minneapolis, MN, USA). In the other cases, the Covidien Endo Clip I ML 10 mm (Covidien/Medtronic, Inc.) was applied to areas of bleeding or any vessels cut by the stapler. The clipping was not considered as method of reinforcement.

The resected stomach specimen was then removed through one of the 13-mm ports. After testing for leaks with methylene blue dye (100 mL), a drain was placed alongside the staple line. All patients underwent an additional test with methylene blue solution on postoperative day 1. If no leakage was detected, an oral diet was resumed. The patients were discharged on postoperative day 2.

### Protocol for Thrombosis Prophylaxis

The protocol for thrombosis prophylaxis included lower extremity compression (LEC) and anticoagulation therapy including subcutaneous (SC) low-molecular-weight heparins (LMWHs). All patients received pre- and postoperative Nadroparin, 3800 Unit, SC. The first dose was administered 12 h before surgery. The second dose was administered 8–12 h after surgery. The LMWHs were maintained up to postoperative day 20.

### Statistical Analysis

Data were analyzed using SAS University Edition (SAS Institute Inc., Cary, NC, USA). To explore the risk factors associated with the primary outcome, a univariate analysis was performed using Student’s *t* test for continuous variables and either Pearson’s chi-square test or Fisher’s exact test for categorical variables. Multiple logistic regression with stepwise variable selection was used to construct a model for prediction of the primary outcome. Backward stepwise elimination and forward stepwise selection were used to build a model. Independent variables that were significantly associated (*p* < 0.2) with the primary outcome in the univariate analysis were entered into the model. Using backward elimination, risk factors (*p* < 0.2) were kept in the model. Forward stepwise selection was then used to find a stable model. The calibration of the model was tested using the Hosmer–Lemeshow goodness-of-fit test. The discriminatory capability of the model was assessed using the c-statistic. The regression equation used to generate the model was used to construct a free online version of the calculator using the Cleveland Clinic Risk Calculator Constructor (http://www.r-calc.com).

## Results

Table 1 presents the baseline characteristics and comorbidities of 552 patients. All patients were Caucasian. The reoperation rate due to HC was 4.02 %. All cases underwent re-laparoscopy and evacuation of hematoma. In cases where the source of bleeding was found, a hemoclip was used to stop it. One patient developed gastric fistula on postoperative day 10. To control the leak, a self-expandable covered stent was placed at the level of gastroesophageal junction for 4 weeks. After removal of the stent, no leak was present. The rest of patients did well after re-laparoscopy.

**Table 1:** Characteristics of patients

Independent variable	Mean (±)/*n* (%)	Definition
Age (years)	41.0 ± 11.6	–
Gender, female	277 (53)	–
BMI (kg/m^2^)	47.3 ± 7.3	–
Diabetes	108 (21)	Diabetes requiring medication.
Hypertension	240 (46)	Hypertension requiring medication.
Dyslipidemia	56 (11)	Dyslipidemia requiring medication.
Obstructive sleep apnea	26 (5)	History of obstructive sleep apnea and continuous positive airway pressure (CPAP).
Hypothyroidism	66 (13)	History of hypothyroidism.
Hyperthyroidism	1 (<1)	History of hyperthyroidism.
Surgical factors	*n* (%)	Definition
Experience	356 (68)	Over 50 bariatric procedures per year.
Qualifications	432 (83)	Completion of residency training in general surgery (6 years) and obtain of certification.
Staple line reinforcement	303 (61)	Oversewing the staple line.

The majority of the bleeding complications in LSG occurred at the staple line (12 cases). We were unable to locate the source of the bleeding in six cases. The bleeding arose from the omentum in only three cases. (Table 2).

**Table 2:** Source of bleeding

Location	*n* (%)
Staple line	12 (57)
Omentum	3 (14)
Unknown	6 (29)
Total	21 (100)

Of the 12 examined variables, four were associated with a risk of HC (Table 2). Protective factors for HC were no history of obstructive sleep apnea (odds ratio [OR], 0.22; 95 % confidence interval [CI], 0.05–0.94) and no history of hypertension (OR, 0.38; 95 % CI, 0.14–1.05). Two factors were associated with a higher risk of HC: a low level of surgical expertise (OR, 2.85; 95 % CI, 1.08–7.53) and no staple line reinforcement (OR, 3.34; 95 % CI, 1.21–9.21) (Table 3).

**Table 3:** Predictive factors of primary outcome based on multivariate analysis

Factor	Adjusted odds ratio	95 % CI	Estimate	Standard error for the estimate
No history of hypertension	0.38	0.14–1.05	−0.486	0.26
No history of obstructive sleep apnea	0.22	0.05–0.90	−0.749	0.36
Low surgeon experience in bariatric surgery	2.85	1.08–7.5	0.524	0.25
No staple line reinforcement	3.34	1.21–9.21	0.603	0.26

The multiple logistic regression equation was as follows:

L = −2.58 + (−0.749* no history of obstructive sleep apnea) + (−0.486* no history of hypertension) + (0.603* no staple line reinforcement) + (0.524* low level of expertise in bariatric surgery).

The model demonstrated good calibration (Hosmer–Lemeshow goodness-of-fit test), χ^2^ = 4.7, *p* = 0.56, and satisfactory discrimination (c-statistic = 0.74). Cross-validation was used to perform the internal validation of the generated model and showed similar performance (c-statistic = 0.67; 95 % CI, 0.54–0.81). The examples of the estimated probability of HC are as follows:

- Estimated risk of HC in a healthy patient who was operated on by an experienced surgeon and the staple line was reinforced by running suture would be 2.19 %.
- Estimated risk of HC in a healthy patient who was operated on by a less experienced surgeon and the staple line was reinforced by running suture would be 3.63 %.
- Estimated risk of HC in a healthy patient who was operated on by an experienced surgeon and without staple line reinforcement would be 4.25 %.
- Estimated risk of HC in a patient with hypertension who was operated on by an experienced surgeon and without staple line reinforcement would be 6.72 %.
- Estimated risk of HC in a healthy patient who was operated on by a less experienced surgeon and without staple line reinforcement would be 6.98 %.
- Estimated risk of HC in a patient with hypertension who was operated by a less experienced surgeon and without staple line reinforcement would be 10.81 %.
- Estimated risk of HC in a patient with hypertension and obstructive sleep apnea who was operated by a less experienced surgeon and without staple line reinforcement would be 20.42 %.

The regression equation was the basis for development of an HC risk calculator called SLEEVE BLEED. A free version of the calculator is available at http://www.r-calc.com under the bariatric surgery section.

## Discussion

The incidence of bleeding following laparoscopic sleeve gastrectomy ranges from 1.16 to 4.94 % [6]. HC are usually the result of staple line bleeding. However, there are other sources of bleeding, such as the omentum, the short gastric vessels, and the abdominal wall [12]. The incidence of bleeding in the present study was 4.02 %.

This novel study presents the first stratification system to estimate the risk of HC after LSG. Currently available risk stratification systems are based on old data of different bariatric procedures and do not include surgical factors [8–10]; they are mainly applicable to gastric bypass.

A recent study published by Aminian et al. [11] analyzed the data of 5871 cases of primary LSG extracted from the American College of Surgeons National Surgical Quality Improvement Program (ACS-NSQIP) database. Several factors that contributed to the risk of serious adverse events were identified as follows: diabetes, body mass index, male sex, congestive heart failure, steroid use, bilirubin level, and hematocrit level. These factors showed moderate discriminative ability (c-statistic = 0.68). The authors developed a calculator specific to LSG [11]. The tool can be very useful in overall risk assessment, decision-making, and determination of the need for preoperative treatment optimization. However, the authors included a wide range of serious adverse events as a primary outcome. Another issue is that the study did not consider the surgeon’s experience.

We used similar statistical methodology to develop our risk model. The model focuses only on HC and is specific to LSG. We were limited by the relatively small number of cases (*n* = 552) and variables (*n* = 12) collected from the medical records. However, we considered surgical factors such as experience and qualifications in the risk model. We also included obstructive sleep apnea in the analysis; this is an important factor that is not available in the ACS-NSQIP.

Of the 12 examined variables, four were associated with a risk of HC. Protective factors for HC were no history of obstructive sleep apnea and no history of hypertension. A low level of surgical expertise in bariatric surgery and the lack of staple line reinforcement were associated with a higher risk of HC. Among all of the variables, a history of obstructive sleep apnea and the lack of staple line reinforcement showed the strongest independent associations with the probability of post-LSG HC.

This is the first study to identify factors associated with the probability of bleeding after LSG. We divided the surgeons according to their level of expertise in bariatric surgery (>50 bariatric laparoscopic procedures per year) and qualifications (certificated surgeon vs. resident). We assumed that certificated surgeons were more skilled than residents in laparoscopic technique. Notably, however, the procedures performed by the residents were supervised by experienced surgeons.

Our data show that the surgeon’s level of expertise in bariatric surgery is essential. This is in contrast to the surgeon’s qualifications, which were not associated with the risk of HC. According to the literature, the technical skill of practicing bariatric surgeons varies widely, and greater skill is associated with fewer postoperative complications and lower rates of reoperation, readmission, and visits to the emergency department [13]. However, surgical skill did not affect either the bleeding rate in the early postoperative period or the postoperative weight loss or resolution of medical comorbidities 1 year after surgery [14].

Staple line reinforcement significantly reduced the likelihood of bleeding. A meta-analysis published by Shikora et al. [6] revealed that the incidence of bleeding was dependent on the reinforcement method. In 33 studies where a running suture was used for reinforcement, the bleeding rate was 2.41 %, while in 25 studies without staple reinforcement, the bleeding rate was 4.94 %.

Hypertension and obstructive sleep apnea were important factors in our analysis. Both conditions are associated with increased peripheral vascular resistance and atherosclerosis, which lead to vascular remodeling [15–19]. We suspect that changes in the vascular histology and increased stiffness of the small vessels may have caused disturbances during stapler firing or ligating by the energy device.

The discriminative ability of our model (c-statistic = 0.74) is satisfactory and higher than that of other reported models [8, 11]. Notably, a cut-off value of 0.7 is needed for good discrimination. Using the regression equation, we developed a risk calculator termed SLEEVE BLEED. The online version of this calculator can help in surgical decision-making process, determination of the need for staple line reinforcement, and choosing which surgeon should do the surgery. This tool can be used as a supplement to the calculator developed by Aminian et al. [11].

### Limitations

This study has several limitations. First, the data were derived from a single high-volume bariatric center. Therefore, the number of patients included in the analysis was limited, and the risk assessment tool may be less accurate than that for other hospitals. In addition, we performed an internal validation because of the limited number of patients. However, the risk model must be validated using external validation. On the other hand, the LSG techniques vary in large multicenter datasets; different types of bougies, different approaches to mobilization of the diaphragmatic crura, and different techniques of suturing are used. Our risk model focuses on the above-described technique, which is reproducible. Additionally, we did not analyze the type of stapler used in the procedure.

It is debatable whether or not cases of bleeding from different sites should be included in an analysis because certain factors predispose patients to certain types of bleeding. For example, reinforcement of the staple line with suture may reduce bleeding from the staple line.

Every patient underwent pulmonary preoperative evaluation. However, the incidence of obstructive sleep apnea in our study is low and may be an underrepresentation. According to Peromna-Haavist et al. [20], 71 % of patients qualified to undergo bariatric surgery fulfilled the diagnostic criteria for obstructive sleep apnea, and about 80 to 90 % of cases of obstructive sleep apnea remained undiagnosed. The small number of patients diagnosed with OSA in our study may be a result of limited access to polysomnography. Thus, all cases should be considered severe. Finally, the data of other important factors such as liver or renal dysfunction and a history of anticoagulant treatment were not included in the analysis.

## Conclusion

This study revealed an association between the risk of HC and four predictive factors: obstructive sleep apnea, hypertension, level of surgeon expertise in bariatric surgery, and reinforcement of the staple line. Our risk assessment model for HC after LSG can contribute to surgical decision-making process. The association between hypertension, obstructive sleep apnea, and postoperative HC should be investigated in further studies.

## References

[1] Angrisani L, Santonicola A, Iovino P (2015). Bariatric surgery worldwide 2013. Obes Surg.

[2] Janik MR, Stanowski E, Paśnik K (2016). Present status of bariatric surgery in Poland. Videosurgery Miniinv.

[3] Brethauer SA, Hammel JP, Schauer PR (2009). Systematic review of sleeve gastrectomy as staging and primary bariatric procedure. Surg Obes Relat Dis.

[4] Schauer PR, Bhatt DL, Kirwan JP (2014). Bariatric surgery versus intensive medical therapy for diabetes—3-year outcomes. N Engl J Med.

[5] Paluszkiewicz R, Kalinowski P, Wróblewski T (2012). Prospective randomized clinical trial of laparoscopic sleeve gastrectomy versus open roux-en-Y gastric bypass for the management of patients with morbid obesity. Videosurgery Miniinv.

[6] Shikora SA, Mahoney CB (2015). Clinical benefit of gastric staple line reinforcement (SLR) in gastrointestinal surgery: a meta-analysis. Obes Surg.

[7] Frezza EE, Reddy S, Gee LL (2009). Complications after sleeve gastrectomy for morbid obesity. Obes Surg.

[8] Finks JF, Kole KL, Yenumula PR (2011). Predicting risk for serious complications with bariatric surgery: results from the Michigan bariatric surgery collaborative. Ann Surg.

[9] Ramanan B, Gupta PK, Gupta H (2012). Development and validation of a bariatric surgery mortality risk calculator. J Am Coll Surg.

[10] DeMaria EJ, Portenier D, Wolfe L (2007). Obesity surgery mortality risk score: proposal for a clinically useful score to predict mortality risk in patients undergoing gastric bypass. Surg Obes Relat Dis.

[11] Aminian A, Brethauer SA, Sharafkhah M (2015). Development of a sleeve gastrectomy risk calculator. Surg Obes Relat Dis.

[12] Jossart GH (2010). Complications of sleeve gastrectomy: bleeding and prevention. Surg Laparosc Endosc Percutan Tech.

[13] Birkmeyer JD, Finks JF, O’Reilly A (2013). Surgical skill and complication rates after bariatric surgery. N Engl J Med.

[14] Scally P, Varban OA, Carlin AM, et al. Michigan bariatric surgery collaborative. Video ratings of surgical skill and late outcomes of bariatric surgery. JAMA Surg. Published online April 132016. 10.1001/jamasurg.2016.0428 PMC5889849 27074114

[15] Park JB, Schiffrin EL (2001). Small artery remodeling is the most prevalent (earliest?) form of target organ damage in mild essential hypertension. J Hypertens.

[16] Kanagy NL (2009). Vascular effects of intermittent hypoxia. ILAR J.

[17] Lévy P, Pépin JL, Arnaud C (2009). Obstructive sleep apnea and atherosclerosis. Prog Cardiovasc Dis.

[18] Feng J, Zhang D, Chen B (2012). Endothelial mechanisms of endothelial dysfunction in patients with obstructive sleep apnea. Sleep Breath.

[19] Alexander RW (1995). Theodore Cooper memorial lecture. Hypertension and the pathogenesis of atherosclerosis. Oxidative stress and the mediation of arterial inflammatory response: a new perspective. Hypertension.

[20] Peromaa-Haavisto P, Tumilehto H, Kossi J (2016). Prevalence of obstructive sleep apnea among patients admitted for bariatric surgery. A prospective multicenter trial. Obes Surg.

